# *O*‐GlcNAcylation Regulates Centrosome Behavior and Cell Polarity to Reduce Pulmonary Fibrosis and Maintain the Epithelial Phenotype

**DOI:** 10.1002/advs.202303545

**Published:** 2023-11-14

**Authors:** Fan Yu, Song Yang, Hua Ni, Dai Heng, Xuemei Wu, Mulin Yang, Xinming Zhang, Yuxin Cao, Yandong Pei, Di An, Dengwen Li, Dayong Liu, Lin Liu, Leiting Pan, Quan Chen, Xueliang Zhu, Jun Zhou

**Affiliations:** ^1^ State Key Laboratory of Medicinal Chemical Biology Haihe Laboratory of Cell Ecosystem Frontiers Science Center for Cell Responses Tianjin Key Laboratory of Protein Science College of Life Sciences Nankai University Tianjin 300071 China; ^2^ School of Health and Life Sciences University of Health and Rehabilitation Sciences Qingdao 266071 China; ^3^ Department of Endodontics and Laboratory of Stem Cells Endocrine Immunology Tianjin Medical University School of Stomatology Tianjin 300070 China; ^4^ State Key Laboratory of Cell Biology CAS Centre for Excellence in Molecular Cell Science Institute of Biochemistry and Cell Biology Shanghai Institutes for Biological Sciences Chinese Academy of Sciences Shanghai 200031 China; ^5^ Center for Cell Structure and Function Shandong Provincial Key Laboratory of Animal Resistance Biology College of Life Sciences Shandong Normal University Jinan 250014 China

**Keywords:** cell polarity, centrosome, microtubule, *O*‐GlcNAcylation, phase separation, pulmonary fibrosis

## Abstract

*O*‐GlcNAcylation functions as a cellular nutrient and stress sensor and participates in almost all cellular processes. However, it remains unclear whether *O*‐GlcNAcylation plays a role in the establishment and maintenance of cell polarity, because mice lacking *O*‐GlcNAc transferase (OGT) are embryonically lethal. Here, a mild *Ogt* knockout mouse model is constructed and the important role of *O*‐GlcNAcylation in establishing and maintaining cell polarity is demonstrated. *Ogt* knockout leads to severe pulmonary fibrosis and dramatically promotes epithelial‐to‐mesenchymal transition. Mechanistic studies reveal that OGT interacts with pericentriolar material 1 (PCM1) and centrosomal protein 131 (CEP131), components of centriolar satellites required for anchoring microtubules to the centrosome. These data further show that *O*‐GlcNAcylation of PCM1 and CEP131 promotes their centrosomal localization through phase separation. Decrease in *O*‐GlcNAcylation prevents PCM1 and CEP131 from localizing to the centrosome, instead dispersing these proteins throughout the cell and impairing the microtubule‐centrosome interaction to disrupt centrosome positioning and cell polarity. These findings identify a previously unrecognized role for protein *O*‐GlcNAcylation in establishing and maintaining cell polarity with important implications for the pathogenesis of pulmonary fibrosis.

## Graphical abstract

In this study, the authors demonstrate the important role of *O*‐GlcNAcylation in establishing and maintaining polarity. OGT deficiency impairs the phase separation of centriolar satellite proteins, disrupts the microtubule‐centrosome interaction, and leads to the loss of cell polarity. These findings identify a previously unrecognized role of *O*‐GlcNAcylation in cell polarity with important implications for the pathogenesis of pulmonary fibrosis.

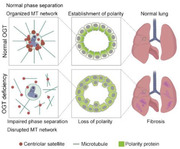

## 1 Introduction

Cell polarity refers to a structurally and functionally asymmetric cellular organization characterized by uneven distribution of cellular constituents, polarized localization of cytoskeleton components, and gradients of soluble molecules or asymmetric distrifbution of attached structures.^[^ ^1^ ^]^ The establishment and maintenance of cell polarity is closely related to the centrosome and its attached microtubules. The centrosome, described as a “polar corpuscle”, has been implicated in the establishment of cell polarity during migration, tissue growth, and tissue homeostasis.^[^ ^2^ ^]^ Cells can establish and reorient their polarity axis through the organization of microtubules anchored at the centrosome.^[^ ^3^ ^]^ Microtubules are intrinsically polarized and contribute to cell polarity through their orientation, density, and post‐translational modifications (PTMs). Once established, the longevity of cell polarity varies and depends on the cell type.^[^ ^4^ ^]^

Polarity must be stable in highly differentiated cells, especially epithelial cells.^[^ ^1^, ^5^ ^]^ Indeed, loss of cell polarity can activate epithelial‐to‐mesenchymal transition (EMT), a pathophysiological process in which the phenotype of epithelial cells changes to a fibroblast‐like mesenchymal phenotype.^[^ ^6^ ^]^ EMT therefore contributes to several disease states, including the invasive and metastatic hallmarks of cancer and tissue fibrosis in fibrotic disorders such as pulmonary fibrosis and liver cirrhosis.^[^ ^7^ ^]^ During EMT, epithelial cells first lose cell polarity before weakening cellular junctions to acquire an invasive phenotype.^[^ ^8^ ^]^ Therefore, the maintenance of epithelial cell polarity helps to resist fibrosis. For example, pulmonary fibrosis is characterized by fibroblast proliferation and extracellular matrix deposition accompanied by inflammatory damage and structural destruction.^[^ ^9^ ^]^ Although pulmonary fibrosis is associated with several etiologies including aging, injury, inflammation, genetic and environmental factors, and bacterial and virus infections, the exact pathogenesis remains uncertain.^[^ ^10^ ^]^ One of the most accepted theories is that pulmonary fibrosis represents an excessive stress response of type II alveolar epithelial cells characterized by frustrated re‐epithelization and fibroblast activation.^[^ ^11^ ^]^

Unlike other glycosylation events occurring outside the cell membrane, *O*‐GlcNAcylation is a special PTM that occurs inside cells and involves the reversible attachment of a single *O*‐linked *N*‐acetylglucosamine (*O*‐GlcNAc) moiety to serine or threonine residues in proteins.^[^ ^12^ ^]^ Two enzymes regulate this modification, *O*‐GlcNAc transferase (OGT) and *O*‐GlcNAcase (OGA), which add or remove GlcNAc from proteins, respectively.^[^ ^13^ ^]^ *O*‐GlcNAcylation is a highly dynamic and rapid response to almost all environmental or physiological stimuli including heat, ultraviolet irradiation, salt, and mechanical damage. Therefore, *O*‐GlcNAcylation is considered a cellular stress sensor.^[^ ^14^ ^]^ Disruption of *O*‐GlcNAcylation homeostasis is implicated in the pathogenesis of many diseases including cancer, diabetes, neurodegeneration, and retinopathy.^[^ ^15^ ^]^ *O*‐GlcNAcylation affects over 70% of cellular proteins and participates in almost all cellular processes.^[^ ^12^, ^16^ ^]^ It is so important that *Ogt* knockout results in embryonic lethality in mice, making the study of the long‐term function of *O*‐GlcNAcylation in development or tissue homeostasis challenging. We hypothesized that *O*‐GlcNAcylation, as the cellular stress sensor, may also participate in the pathogenesis of pulmonary fibrosis and the regulation of cell polarity.^[^ ^17^ ^]^ Here we demonstrate that *O*‐GlcNAcylation regulates cell polarity by modifying pericentriolar material 1 (PCM1) and centrosomal protein 131 (CEP131), centriolar satellite components responsible for anchoring microtubules to the centrosome. *O*‐GlcNAcylation is critical for the localization of PCM1 and CEP131 to the centrosome by regulating their phase separation. Deficiency in *O*‐GlcNAcylation disperses PCM1 and CEP131, impairs the connections between microtubules and the centrosome, and disturbs cell polarity, which could be an underlying mechanism of pulmonary fibrosis.

## 2 Results

### 2.1 *Ogt* Inducible Knockout Mice Develop Severe Pulmonary Fibrosis

**Figure 1:**
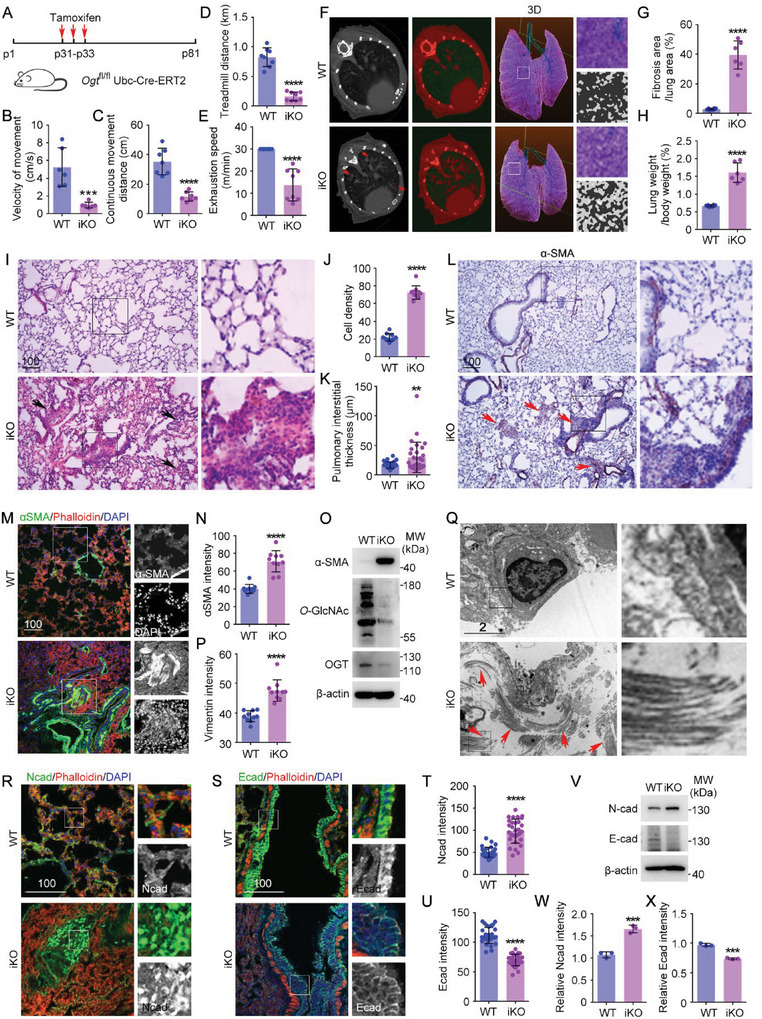
*Ogt iKO* mice develop severe pulmonary fibrosis. A) *Ogt^fl/fl^ Ubc‐Cre‐ERT2* mice were administered tamoxifen daily by intraperitoneal injection for three consecutive days (P31–P33) and then bred normally for 50 days to obtain “mild” induced *Ogt* iKO mice. B–E) The physical ability of *Ogt* iKO mice was assessed, including their speed (B, *n* = 6 mice) and continuous distance of each natural movement (C, *n* = 7 mice). The physical ability was further assessed on a treadmill (D,E, *n* = 8 mice). F,G) Micro‐CT imaging was performed to examine the cardiopulmonary status of mice (F). The percentage of fibrosis area in the lung was quantified (G, *n* = 8 mice). H) Lungs isolated from WT and *Ogt* iKO mice were weighed, and the percentage of lung weight to body weight was quantified. *n* = 8 mice. I–K) Hematoxylin and eosin staining of formalin‐fixed, paraffin‐embedded tissue sections from the lung (I) and quantification of the cell density (J, *n* = 10 fields) and pulmonary interstitial thickness (K, *n* = 20 fields). L) Lung sections from WT and *Ogt* iKO mice were subjected to immunohistochemical staining with antibodies targeting α‐SMA. M,N) Lung sections from WT and *Ogt* iKO mice were subjected to immunofluorescence microscopy with antibodies targeting α‐SMA. The fluorescent intensity of α‐SMA was quantified. *n* = 10 fields. O) Lungs isolated from WT and *Ogt* iKO mice were lysed and subjected to immunoblotting with the indicated antibodies. P) Lung sections from WT and *Ogt* iKO mice were subjected to immunofluorescence microscopy with antibodies targeting vimentin, and the fluorescence intensity of vimentin was quantified. *n* = 10 fields. Q) Lungs isolated from WT and *Ogt* iKO mice were subjected to TEM. Collagen was indicated with red arrows. R–U) Lung sections from WT and *Ogt* iKO mice were subjected to immunofluorescence microscopy with antibodies targeting N‐cadherin (R) and E‐cadherin (S). The fluorescent intensity of N‐cadherin (T, *n* = 20 fields) and E‐cadherin (U, *n* = 20 fields) was quantified. V–X) Lung tissue lysates from WT and *Ogt* iKO mice were subjected to immunoblotting with the indicated antibodies. The relative intensity of N‐cadherin (W) and E‐cadherin (X) was quantified. All experiments were repeated at least three times. Scale bars are 10 unless specifically indicated (µm). ^**^ *p* < 0.01, ^***^ *p* < 0.001, ^****^ *p* < 0.0001. Error bars indicate SD.

As one of the most common PTMs, *O*‐GlcNAcylation participates in almost every signaling pathway and cellular process. Therefore, *Ogt* knockout is embryonically lethal, and *Ogt* KO mouse embryos die at E4.5. In adult mice, inducible *Ogt* knockout also leads to death after 2–4 weeks. Therefore, to investigate OGT function in tissue homeostasis, we first generated a mild *Ogt* inducible knockout (iKO) mouse model with low‐dose tamoxifen administered for only three days (**Figure** **1**A). These mice lived for another 2–3 months after tamoxifen injection. After 50 days of *Ogt* iKO, the mice developed bradykinesia, with much slower movements and shorter distances of continuous movement (Figure 1B,C). We then assessed their physical activity on a treadmill and found a significant decrease in activity in *Ogt* iKO mice compared with wildtype controls (Figure 1D,E; Figure S1A,B, Supporting Information).

We next performed micro‐computed tomographic (micro‐CT) imaging to examine the cardiopulmonary status of *Ogt* iKO mice (Figure 1F–H), which revealed increased lung tissue density consistent with severe fibrosis. The area of lung fibrosis was significantly greater in *Ogt* iKO mice compared with controls (Figure 1G), and the percentage of lung weight to body weight was also significantly increased due to abnormal tissue proliferation (Figure 1H). Histopathological examination of lung tissue sections confirmed severe pulmonary fibrosis and morphological abnormalities in *Ogt* iKO mice. There was increased tissue density along with septal thickening, masson trichrome staining revealed increased collagen deposition (Figure 1I–K; Figure S1C, Supporting Information), and immunohistochemical staining showed accumulation of α‐SMA‐positive myofibroblasts in *Ogt* iKO mice, suggesting extensive tissue remodeling and fibrosis (Figure 1L). These observations were confirmed by immunofluorescence imaging and immunoblotting: α‐SMA expression was significantly increased in *Ogt* iKO mice (Figure 1M–P). Consistent with this, transmission electron microscopy (TEM) imaging showed massive fibrosis in *Ogt* iKO mouse lungs (Figure 1Q).

To further explore the effect of *Ogt* iKO on epithelial remodeling, we examined EMT, which is implicated in fibrosis. Immunofluorescence imaging revealed an abnormal increase in N‐cadherin (N‐cad) in *Ogt* iKO lungs, while E‐cadherin (E‐cad) expression was decreased in alveolar epithelial cells (Figure 1R–U). Moreover, the shape and arrangement of alveolar epithelial cells were also altered in *Ogt* iKO mice: cells were longer and multilayered compared with wild‐type cells, suggesting EMT (Figure 1S). Immunoblotting analysis confirmed an increase in E‐cad and a decrease in N‐cad in *Ogt* iKO mouse lungs (Figure 1V–X).

We also used MCF10A cells to confirm the effect of *O*‐GlcNAcylation levels on EMT. Cells were treated with OSMI‐1, a widely used specific OGT inhibitor, to decrease *O*‐GlcNAcylation, with thiamet G (TMG), an inhibitor of OGA, used as a comparison. While there was no overt morphological evidence of EMT with the short‐term treatment of cells with TGF‐β, co‐treatment with OSMI‐1 elongated cells, the spindle shape suggesting EMT (Figure S1E–G, Supporting Information). Confirming this, immunoblot analysis revealed an increase in N‐cad and a decrease in E‐cad in OSMI‐1‐treated cells (Figure S1H–J, Supporting Information). Together, these results suggest that decreased *O*‐GlcNAcylation could accelerate EMT, thus promoting fibrosis.

### 2.2 Inhibition of *O*‐GlcNAcylation Disrupts Cell Polarity

**Figure 2:**
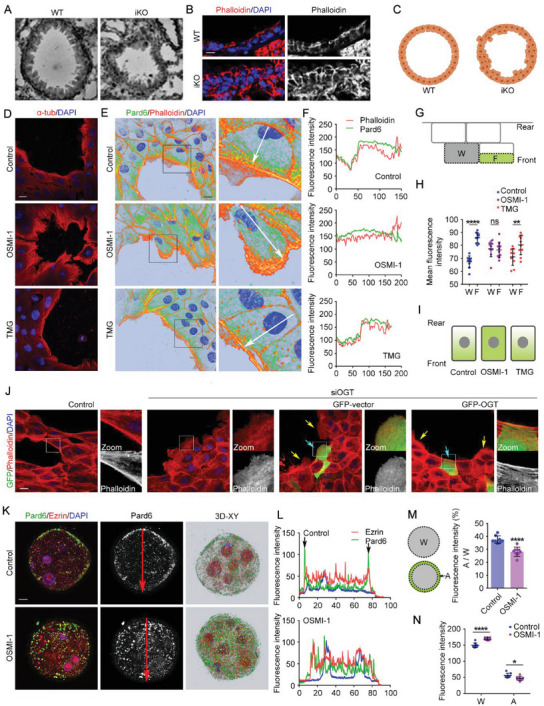
Inhibition of *O*‐GlcNAcylation disrupts cell polarity. A–C) Lung sections from WT and *Ogt iKO* mice were subjected to microscopy to visualize the organization of pulmonary epithelial cells. D) MCF10A cells were treated with DMSO (Control), OSMI‐1, or TMG for 24 h and then subjected to immunofluorescence microscopy with antibodies targeting tubulin. E–I) MCF10A cells were treated with DMSO (Control), OSMI‐1, or TMG for 24 h and then subjected to immunofluorescence microscopy with antibodies targeting Pard6 and phalloidin. The fluorescence intensity of Pard6 and phalloidin across the cell along the white arrow in panel E was assessed using ImageJ (F). The fluorescence intensity of Pard6 in each cell and the front area was quantified. *n* = 10 cells. W, Whole; F, Front (G–I). J) MCF10A cells were transfected with control or OGT siRNA for 24 h and then transfected with GFP‐vector or GFP‐OGT plasmids for another 24 h, as indicated. The cells were subjected to immunofluorescence microscopy with phalloidin. Cells successfully transfected with plasmids were indicated by blue arrows, while cells without transfection were indicated by yellow arrows. K–N) Mouse embryos were cultured in vitro and treated with DMSO (Control) or OSMI‐1 at the 8‐cell stage for 12 h. Embryos were then subjected to immunofluorescence microscopy with antibodies targeting Pard6 and Ezrin (K). The fluorescence intensity across the embryo along the red arrow was assessed using ImageJ (L). The fluorescence intensity of Pard6 in each embryo and in the apical area was quantified. *n* = 10 embryos. W, Whole; A, Apical (M,N). All experiments were repeated at least three times. Scale bars, 10 µm. ^*^ *p* < 0.05, ^**^ *p* < 0.01, ^****^ *p* < 0.0001. Error bars indicate SD.

We reasoned that the observed fibrosis and EMT might be due to disrupted polarity, since the epithelial cell arrangement was severely disordered in *Ogt* iKO mice (**Figure** **2**A–C). Therefore, we tested the effect of *O*‐GlcNAcylation on polarity in several different models. First, we used MCF10A cells to examine front‐rear polarity. MCF10A cells were plated on Matrigel, where they spontaneously formed small cavities with smooth edges. However, when the *O*‐GlcNAcylation level was decreased by OSMI‐1 treatment or OGT knockdown, the cavities became disordered with rough edges (Figure 2D; Figure S2A,B, Supporting Information). Consistent with this morphology, OSMI‐1 altered the localization of Pard6, a polarity indicator: Pard6 accumulated along the edges close to the cavities in control cells, while OSMI‐1 treatment induced Pard6 dispersion throughout cells without any accumulation along the front edges. We also treated cells with TMG. While OSMI‐1 significantly disrupted polarity in MCF10A cells, increasing *O*‐GlcNAcylation with TMG had no significant effect (Figure 2E–I). We also examined the localization of Ezrin but found no obvious changes upon OSMI‐1 treatment (Figure S2C, Supporting Information). Of note, OSMI‐1 treatment slightly increased Pard6 protein expression, probably due to a feedback mechanism compensating by increasing Pard6 expression to rescue polarity (Figure S2D, Supporting Information). These effects caused by OGT knockdown could be rescued by its overexpression. Overexpression of OGT reconstructed cell polarity and the smooth edges of small cavities, compared to the cells with GFP‐vector overexpression or cells without OGT overexpression (Figure 2J). We then confirmed the impact of *O*‐GlcNAcylation on front‐rear polarity using migrating RPE1 cells. The cells kept the same migration direction and speed and therefore formed a smooth edge during the migration, which was disrupted after OSMI‐1 treatment (Figure S2E, Supporting Information).

We next examined the effect of *O*‐GlcNAcylation on apical‐basal polarity. Mouse embryos begin to establish polarity at the 8‐cell stage. When treated with OSMI‐1 at the 8‐cell stage, Pard6 could not localize to the apical pole but spread throughout the cell, suggesting a failure of establishment of apical‐basal polarity (Figure 2K–N). Taken together, these results suggest a critical role for *O*‐GlcNAcylation in the establishment and maintenance of both front‐rear and apical‐basal polarity.

### 2.3 Inhibition of *O*‐GlcNAcylation Disturbs Centrosome Positioning and the Microtubule‐Centrosome Interaction

**Figure 3:**
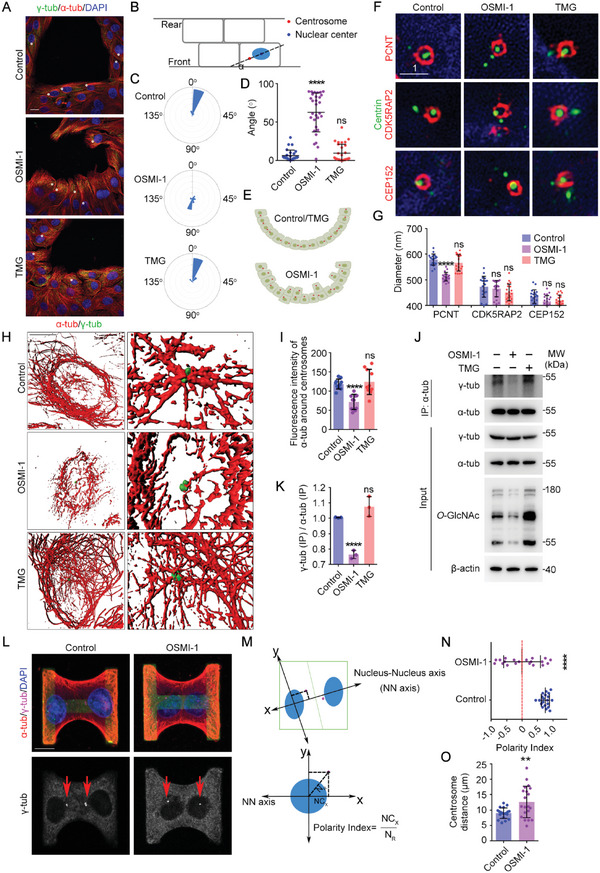
Inhibition of *O*‐GlcNAcylation disturbs centrosome positioning and the microtubule‐centrosome interaction. A–E) MCF10A cells were treated with DMSO (Control), OSMI‐1, or TMG for 24 h and then subjected to immunofluorescence microscopy with antibodies targeting α‐tubulin and γ‐tubulin. Centrosomes are highlighted with white circles (A). The nucleus‐centrosome axis of each cell along the edge of the cavity was quantified using ImageJ (B–E). The angle (α) between the cavity edge and the nucleus‐centrosome axis of each cell was measured as indicated in panel B and was shown in panel D; when α > 90°, the angle = 180° – α. *n* = 30 cells. F,G) MCF10A cells were treated with DMSO (Control), OSMI‐1, or TMG for 24 h and then subjected to immunofluorescence microscopy with the indicated antibodies (F). The diameter of each ring was quantified using ImageJ (G, *n* = 30 cells). H,I) MCF10A cells were treated with DMSO (Control), OSMI‐1, or TMG for 24 h and then subjected to immunofluorescence microscopy with antibodies targeting α‐tubulin and γ‐tubulin (H). The fluorescence intensity of α‐tubulin around the centrosome was assessed using ImageJ (I, *n* = 10 cells). J,K) MCF10A cells were treated with DMSO (Control), OSMI‐1, or TMG for 24 h and cell lysates were immunoprecipitated with antibodies targeting α‐tubulin and immunoblotted with the indicated antibodies. L–O) MCF10A cells were plated on H‐shaped micropatterns and fixed after one cell division to form daughter‐cell doublets. Cells were then subjected to immunofluorescence microscopy with antibodies targeting α‐tubulin and γ‐tubulin (L). The polarity index of each daughter‐cell doublet was assessed as described in the main text (M,N). The distance between the centrosomes of each daughter‐cell doublet was assessed using ImageJ (O). *n* = 30 cells. All experiments were repeated at least three times. Scale bars are 10 unless specifically indicated (µm). ^**^ *p* < 0.01, ^***^ *p* < 0.001, ^****^ *p* < 0.0001; ns, not significant. Error bars indicate SD.

We next sought to understand the mechanism underlying how *O*‐GlcNAcylation affects polarity. Centrosome repositioning has been proven to play a critical role in the establishment of polarity and EMT, so we examined the effects of *O*‐GlcNAcylation on centrosome positioning. Centrosomes in MCF10A cells were localized next to nuclei, resulting in a nucleus‐centrosome axis oriented along the edge of the cavity and a smooth cavity edge. After OSMI‐1 treatment or OGT knockdown, centrosomes were positioned more randomly, further from the nuclei, and closer to the cavity. The disrupted centrosome positioning might therefore explain the disturbed polarity (**Figure** **3**A–E; Figure S3A, Supporting Information).

How did *O*‐GlcNAcylation determine centrosome positioning? We first examined the centrosome structure using different markers and found that pericentriolar material (PCM), the outer layer of the centrosome, was impaired when *O*‐GlcNAcylation levels decreased. PCNT rings after OSMI‐1 treatment or OGT knockdown were obviously smaller, while other centrosome markers showed no significant changes (Figure 3F,G; Figure S3B,C, Supporting Information). PCM is responsible for the connection of centrosomes to microtubules, and further investigation revealed that OSMI‐1 treatment disrupted the microtubule network around centrosomes. Compared with the dense microtubules observed around centrosomes in control cells, there were significantly fewer microtubules attached to centrosomes after OSMI‐1 treatment (Figure 3H,I; Figure S3D, Supporting Information). This result could be confirmed in another cell line. Knockdown or inhibition of OGT also resulted in a disrupted microtubule network and less connection with centrosomes in U2OS cells (Figure S3E,F, Supporting Information). To confirm this finding, we detected a significant decrease in α‐tubulin and γ‐tubulin interactions after OSMI‐1 treatment, indicating a weaker connection between centrosomes and microtubules (Figure 3J,K). Furthermore, PCM functions as the organization center of microtubules, so we examined the effects of *O*‐GlcNAcylation on the reassembly of the microtubule network after complete disassembly. In the control group, the centrosome acted as a single assembly center and microtubules radiated perfectly around the centrosome. However, OSMI‐1 treatment resulted in no prominent organization center but several centers, suggesting that the connections between centrosomes and microtubules were impaired (Figure S3G,H, Supporting Information).

We next used micropatterned cells to confirm disrupted centrosome positioning. Single MCF10A cells were plated on H‐shaped micropatterns and fixed after one cell division. The centrosomes of the daughter‐cell doublets always displayed marked polarization toward the intracellular junction. However, cells treated with OSMI‐1 displayed the opposite polarity orientation. The polarity reversal was quantified by measuring the centrosome x coordinate along the nucleus‐nucleus axis, referred to as the cell polarity index. The negative index and longer distance between the centrosomes of daughter‐cell doublets indicated disrupted centrosome positioning and polarity reversal after OSMI‐1 treatment (Figure 3L–O). Together, these data show that decreased *O*‐GlcNAcylation impairs PCM structure, thus disconnecting microtubules and centrosomes to disrupt centrosome positioning and cell polarity.

### 2.4 PCM1 and CEP131 Are Substrates of *O*‐GlcNAcylation

**Figure 4:**
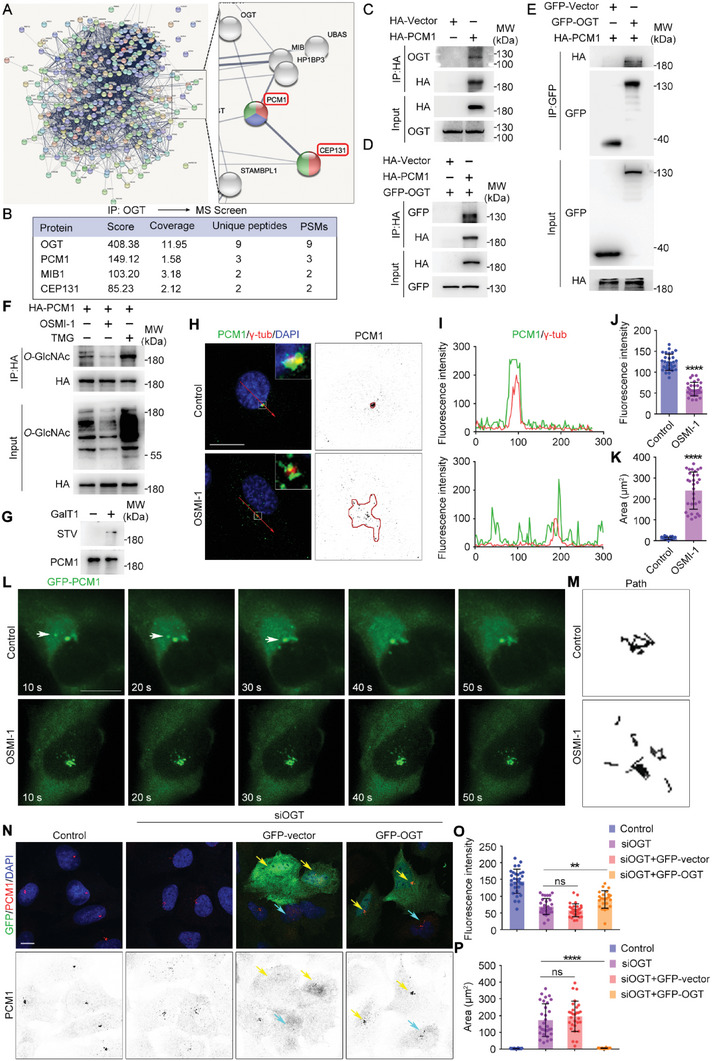
PCM1 and CEP131 are substrates of *O*‐GlcNAcylation. A,B) Lysate from MCF10A cells was immunoprecipitated with OGT antibody and assessed by mass spectrometry. The identified proteins were analyzed using GO analysis on the STRING database. C–E) 293T cells were transfected with the indicated plasmids and subjected to immunoprecipitation. F) 293T cells were transfected with HA‐PCM1 plasmids and treated with DMSO, OSMI‐1, or TMG for 24 h. Cell lysates were subjected to immunoprecipitation. G) Cell lysates from MCF10A cells were immunoprecipitated with antibodies targeting PCM1 and subsequently labeled with GalNAz and biotin, which were then probed with STV‐HRP and antibodies targeting PCM1. H–K) MCF10A cells were treated with DMSO (Control) or OSMI‐1 for 24 h and then subjected to immunofluorescence microscopy with antibodies targeting PCM1 and γ‐tubulin (H). The fluorescence intensity of PCM1 and γ‐tubulin along the red arrow in panel H was assessed using ImageJ (I). The fluorescence intensity of PCM1 around the centrosome (J, *n* = 30 cells) and the distribution area of PCM1 (K, *n* = 30 cells) was quantified using ImageJ. L,M) MCF10A cells were transfected with GFP‐PCM1 plasmid for 24 h, treated with DMSO (Control) or OSMI‐1 for 30 min, and then subjected to live cell imaging (L). The moving paths of GFP‐PCM1 were assessed and quantified using ImageJ (M). N–P) MCF10A cells were transfected with control or OGT siRNA for 24 h and then transfected with GFP‐vector or GFP‐OGT plasmids for another 24 h, as indicated. The cells were subjected to immunofluorescence microscopy with antibody targeting PCM1. Cells successfully transfected with plasmids were indicated by yellow arrows, while cells without plasmids transfection were indicated by blue arrows (N). The fluorescence intensity of PCM1 around the centrosome (O, *n* = 30 cells) and the distribution area of PCM1 (P, *n* = 30 cells) was quantified using ImageJ. All experiments were repeated at least three times. Scale bars, 10 µm. ^****^ *p* < 0.0001. Error bars indicate SD.

We next sought downstream substrates of *O*‐GlcNAcylation. Mass spectrometry analysis identified a series of OGT‐interacting proteins related to PCM. As PCM1 and CEP131 are both centriolar satellite components required to anchor microtubules to the centrosome, we reasoned that they could be the key substrates of *O*‐GlcNAcylation (**Figure** **4**A,B). Immunoprecipitation analysis revealed an interaction between OGT and PCM1 with both exogenous and endogenous proteins (Figure 4C–E). Moreover, PCM1 was *O*‐GlcNAcylated in cells and its *O*‐GlcNAcylation level could be regulated by inhibition or knockdown of OGT/OGA (Figure 4F; Figure S4A, Supporting Information). The *O*‐GlcNAcylation of PCM1 was confirmed by chemoenzymatic labeling of PCM1 by click reaction (Figure 4G). We investigated the effect of *O*‐GlcNAcylation on PCM1, which showed distinct localization when *O*‐GlcNAcylation decreased. In control cells, PCM1 accumulated around the centrosomes, while in OGT‐inhibited/knockdown cells, PCM1 did not localize near the centrosomes but was dispersed throughout the cell (Figure 4H–K; Figure S4B–E, Supporting Information). We quantified the fluorescence intensity of PCM1 around the centrosome and found a significant decrease in PCM1 signal after OSMI‐1 treatment or OGT knockdown (Figure 4H,J; Figure S4B,D, Supporting Information). We also analyzed the distribution of PCM1 in the cells and found that PCM1 was much more dispersed when *O*‐GlcNAcylation decreased (Figure 4H–K; Figure S4B–E, Supporting Information). This result was confirmed in another cell line (Figure S4F,G, Supporting Information). The scattered distribution might be due to impaired PCM1 transport and, consistent with this, live cell imaging showed that after 30 min of OSMI‐1 treatment, PCM1 could no longer be transported to the centrosomes (Figure 4L,M). The impact of OGT knockdown on PCM1 localization could be rescued by OGT overexpression. While PCM1 was dispersed after OGT knockdown and GFP‐vector showed no effects, overexpression of OGT efficiently led to the accumulation of PCM1 around the centrosomes (Figure 4N–P).

### 2.5 *O*‐GlcNAcylation Regulates the Phase Separation of PCM1

**Figure 5:**
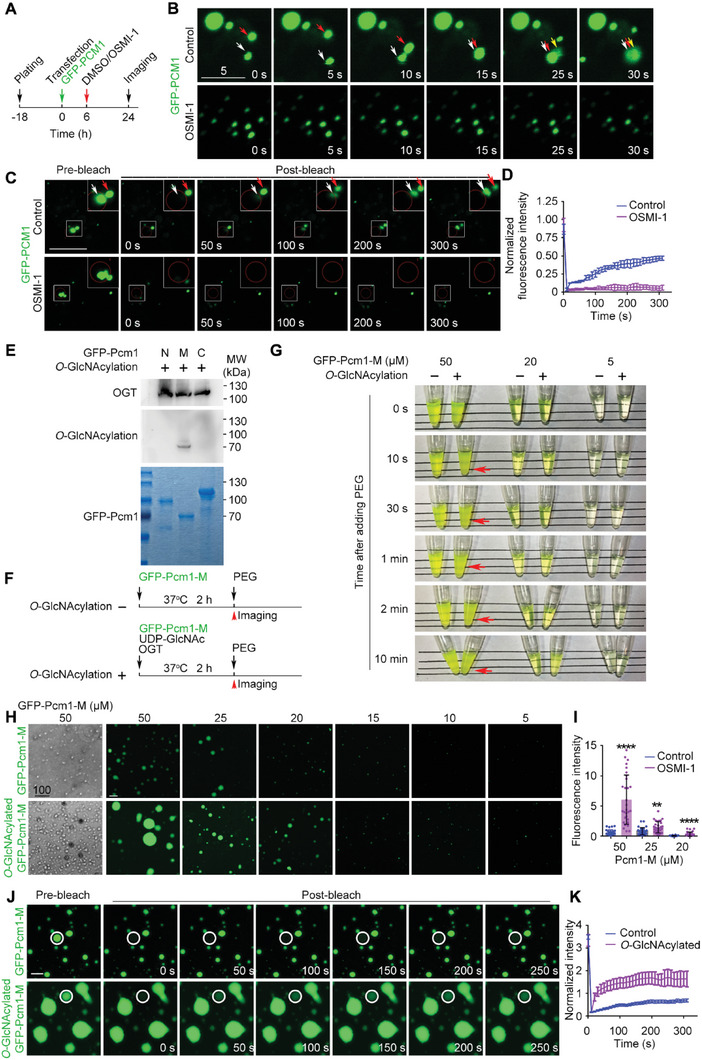
*O*‐GlcNAcylation promotes the phase separation of PCM1. A,B) MCF10A cells were transfected with GFP‐PCM1 plasmid, treated with DMSO (Control) or OSMI‐1 for 18 h, and then subjected to live cell imaging. C,D) MCF10A cells were transfected with GFP‐PCM1 plasmid and treated with DMSO (Control) or OSMI‐1 for 18 h. The GFP‐positive condensates were photobleached followed by live imaging (C), and the fluorescence recovery curve was derived from the automatically quantified intensity of the bleached area (D). E) Three fragments of Pcm1 containing predicted disordered regions were expressed in *E. coli* as His‐GFP‐tagged fusion proteins and purified. In vitro *O*‐GlcNAcylation assay of purified proteins was performed and the reaction mixtures were subjected to immunoblotting with the indicated antibodies. F–K) Purified His‐GFP‐Pcm1M was diluted to the indicated concentrations and an in vitro *O*‐GlcNAcylation assay was performed with or without UDP‐GlcNAc and OGT (F). The mixtures were then treated with PEG and imaged (G,H), and the fluorescence intensity of each droplet was quantified using ImageJ (I, *n* = 30 fields). The GFP‐positive condensates were photobleached followed by live imaging (J), and the fluorescence recovery curve was derived from the automatically quantified intensity of the bleached area (K). All experiments were repeated at least three times. Scale bars are 10 unless specifically indicated (µm). ^**^ *p* < 0.01, ^****^ *p* < 0.0001. Error bars indicate SD.

We next tried to establish the molecular mechanism by which *O*‐GlcNAcylation regulates PCM1 accumulation. PCM1 has recently been reported to be able to assemble through phase separation, so we tested the effect of *O*‐GlcNAcylation on the efficiency of its phase separation. GFP‐PCM1 formed large foci in cells. When two smaller cores met, they fused into a larger one, suggesting liquid‐like properties. However, there were no fused foci or fusing activities in cells treated with OSMI‐1, suggesting that phase separation of PCM1 might be inhibited by decreased *O*‐GlcNAcylation (**Figure** **5**A,B). Fluorescence recovery after photobleaching (FRAP) assays revealed significantly reduced recovery after photobleaching upon OSMI‐1 treatment (Figure 5C,D), suggesting the relatively slower exchange of GFP‐PCM1 between condensates with decreased *O*‐GlcNAcylation.

To confirm the *O*‐GlcNAcylation domain of Pcm1, we purified fragments of Pcm1 from *E. coli* (Pcm1‐N, Pcm1‐M, Pcm1‐C) and found that only Pcm1‐M was abundantly *O*‐GlcNAcylated (Figure 5E). We then examined the effect of *O*‐GlcNAcylation on the phase separation of Pcm1‐M. When Pcm1‐M was *O*‐GlcNAcylated in vitro, phase separation into liquid droplets following the addition of PEG was much quicker than when not *O*‐GlcNAcylated, with the droplet size and number positively correlated with protein concentration (Figure 5F–I; Figure S5A, Supporting Information). Droplets fused rapidly into larger ones in time‐lapse imaging (Figure S5B, Supporting Information), confirming their liquid‐like properties. Strikingly, these *O*‐GlcNAcylated Pcm1‐M proteins formed significantly larger aggregates after several hours, vividly imitating the accumulation of PCM1 around the centrosome inside cells (Figure S5C, Supporting Information). Furthermore, FRAP assays revealed more dynamic protein exchanges between droplets and the milieu when Pcm1‐M was *O*‐GlcNAcylated (Figure 5J,K). Taken together, the *O*‐GlcNAcylation of PCM1 determines its localization around the centrosomes by regulating its domain‐specific phase separation.

### 2.6 CEP131 Is Another *O*‐GlcNAcylation Substrate

**Figure 6:**
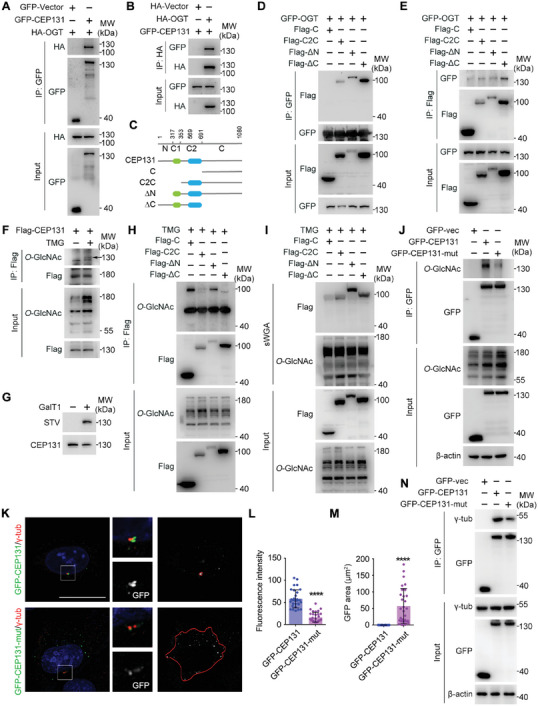
CEP131 is an *O*‐GlcNAcylated protein. A,B) 293T cells were transfected with the indicated plasmids and subjected to immunoprecipitation. C–E) 293T cells were transfected with the indicated plasmids and subjected to immunoprecipitation. F) 293T cells were transfected with Flag‐CEP131 and treated with or without TMG for 24 h and then subjected to immunoprecipitation. G) Cell lysates from MCF10A cells were immunoprecipitated with an antibody targeting CEP131 and subsequently labeled with GalNAz and biotin, which were then probed with STV‐HRP and an antibody targeting CEP131. H,I) 293T cells were transfected with the indicated plasmids and subjected to immunoprecipitation (H) or sWGA pulldown assay (I). J) 293T cells were transfected with GFP‐vector, GFP‐CEP131, or GFP‐CEP131‐mutant, treated with TMG for 24 h, and subjected to immunoprecipitation. K–M) MCF10A cells were transfected with GFP‐CEP131 or GFP‐CEP131‐mutant plasmids for 24 h, and then subjected to immunofluorescence microscopy with antibodies targeting γ‐tubulin (K). The fluorescence intensity of GFP around the centrosome (L, *n* = 30 cells) and the distribution area of GFP (M, *n* = 30 cells) was quantified by ImageJ. N) 293T cells were transfected with the indicated plasmids and subjected to immunoprecipitation. All experiments were repeated at least three times. Scale bars, 10 µm. ^****^ *p* < 0.0001. Error bars indicate SD.

Similar examinations were performed with CEP131. Immunoprecipitation analysis revealed an interaction between OGT and CEP131 (**Figure** **6**A,B). To identify the *O*‐interaction domains and GlcNAcylated sites of CEP131, we first constructed domain‐deleted CEP131 mutants. Co‐immunoprecipitation analysis demonstrated that the Flag‐∆N truncation had the highest affinity interaction with OGT, while Flag‐C had no interaction with OGT, suggesting that the C‐terminal region of CEP131 does not interact with OGT. Even at the lowest expression level, Flag‐∆N still interacted strongly with OGT, suggesting that the N‐terminal region of CEP131 is not the main interaction region with OGT. The interaction between Flag‐C2C and OGT was not as strong as Flag‐∆N, indicating that C1, the first coiled‐coil domain, is the main OGT interaction domain, with C2 also required for the interaction between CEP131 and OGT (Figure 6C–E).

Moreover, CEP131 was *O*‐GlcNAcylated in cells and its *O*‐GlcNAcylation level could be regulated by inhibition or knockdown of OGT/OGA (Figure 6F; Figure S6A, Supporting Information). The *O*‐GlcNAcylation of CEP131 was confirmed by chemoenzymatic labeling of CEP131 by click reaction (Figure 6G). Co‐immunoprecipitation and sWGA pulldown analysis demonstrated that C1 was the main *O*‐GlcNAcylated domain, and C2 was also *O*‐GlcNAcylated at a relatively lower level (Figure 6H,I). We predicted the possible *O*‐GlcNAcylation sites in the C1 region and constructed a single‐site mutant of these sites. All five mutants showed lower *O*‐GlcNAcylation levels, indicating that all five sites were *O*‐GlcNAcylated (Figure S6B, Supporting Information). We then constructed the five‐site mutant of CEP131 and found that the mutant produced much lower *O*‐GlcNAcylation (Figure 6J). Furthermore, the CEP131 mutants all localized differently: while wildtype CEP131 was localized to centrosomes, CEP131 mutants spread throughout the cells (Figure 6K–M). As a result, the interaction between CEP131 mutants and centrosomes (γ‐tubulin) also decreased (Figure 6N), suggesting that *O*‐GlcNAcylation regulates the localization of CEP131 to centrosomes.

To confirm this result, OMSI‐1 was used to decrease *O*‐GlcNAcylation. In control cells, CEP131 accumulated around the centrosomes, while CEP131 proteins were dispersed throughout the cell after OSMI‐1 treatment (Figure S6C–F, Supporting Information). Live cell imaging revealed that upon OSMI‐1 treatment, CEP131 could not be transported to the centrosomes (Figure S6G,H, Supporting Information). The impact of *O*‐GlcNAcylation on CEP131 distribution was confirmed in another cell line (Figure S6I,J, Supporting Information). Moreover, the effect of OGT knockdown on CEP131 localization could be rescued by OGT overexpression. While CEP131 was dispersed after OGT knockdown and GFP‐vector showed no effects, overexpression of OGT rescued the accumulation of CEP131 around the centrosomes (Figure S6K, Supporting Information). Taken together, CEP131 is also *O*‐GlcNAcylated like PCM1. *O*‐GlcNAcylation regulates the localization of CEP131 and PCM1, recruiting them to centrosomes. When *O*‐GlcNAcylation is decreased, PCM1 and CEP131 cannot localize to centrosomes efficiently, leading to the disconnection of microtubules and centrosomes and thus polarity disturbances.

## 3 Discussion

There have been considerable advances in our understanding of *O*‐GlcNAcylation over the past decade, especially in its biochemistry, molecular and cell biology, and physiology.^[^ ^18^ ^]^ Disruption of *O*‐GlcNAc homeostasis is implicated in the pathogenesis of diverse human diseases including cancer, diabetes, and neurodegeneration.^[^ ^19^ ^]^ However, the significance of *O*‐GlcNAcylation in long‐term tissue homeostasis and pathophysiology is only now beginning to be understood.

**Figure 7:**
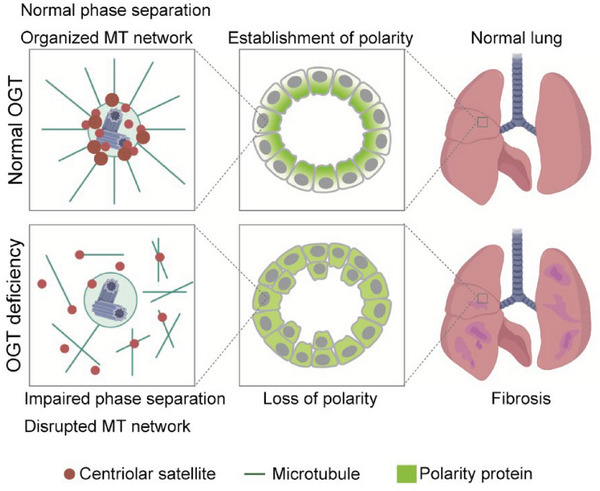
Model for the role of *O*‐GlcNAcylation in epithelial remodeling by regulating the establishment and maintenance of cell polarity.

In this study, we constructed a special *Ogt* knockout model to investigate the long‐term effect of OGT deficiency and found that *Ogt* knockout mice developed severe pulmonary fibrosis. The pathogenesis of pulmonary fibrosis is still incompletely understood, and there is no curative treatment for this disease beyond symptomatic treatment or, in severe cases, lung transplant.^[^ ^20^ ^]^ Therefore, there is an urgent clinical need to explore the underlying mechanisms and to develop new therapeutics. We found that decreased *O*‐GlcNAcylation impaired the connections between microtubules and centrosomes, thus disrupting cell polarity and promoting EMT, eventually leading to fibrosis (**Figure** **7**). Our finding proved an important role for *O*‐GlcNAcylation in the establishment and maintenance of polarity to prevent fibrosis. However, questions remain about the practical application of this finding, including whether *O*‐GlcNAcylation is decreased in clinical fibrosis caused by inflammation or viral infections such as SARS‐CoV‐2 and whether increasing *O*‐GlcNAcylation alleviates fibrosis.

It is interesting to note that, compared with unmodified proteins, increased *O*‐GlcNAcylation dramatically promoted phase separation of purified PCM1. However, the effect of *O*‐GlcNAcylation on phase separation seems to be protein‐dependent.^[^ ^21^ ^]^ It has been reported that *O*‐GlcNAcylation can inhibit the phase separation of some proteins (e.g., SynGAP/PSD‐95).^[^ ^22^ ^]^ We assume that this difference is due to the influence of *O*‐GlcNAcylation on protein configuration, either positive or negative, for phase separation.

We found that PCM1 and CEP131, components of centriolar satellites, were *O*‐GlcNAcylated, and furthermore, their localization around the centrosomes was determined by *O*‐GlcNAcylation. Loss of *O*‐GlcNAcylation prevented them from localizing to the centrosome but led to their cellular dispersal, thus disrupting the connection between microtubules and centrosomes and leading to disordered centrosome positioning and cell polarity. However, it is reasonable to assume that PCM1 and CEP131 are not the only substrates of *O*‐GlcNAcylation in this process. Mass spectrometry results have shown several other related proteins that might also participate in cell polarity. While PCM1 and CEP131 showed the most obvious changes upon decreased *O*‐GlcNAcylation, other possible substrates need to be verified or excluded. With growing knowledge about *O*‐GlcNAcylation and improvements in genetic and pharmacological tools and technologies, we expect further progress in our understanding of this modification in the near future.

## 4 Experimental Section

### Mice

*Ogt*‐floxed mice on a C57BL/6 background were obtained from the Jackson Laboratory (Bar Harbor, ME). Ubc‐Cre‐ERT2 mice were kindly provided by Professor Wen Ning (Nankai University). To obtain OGT‐induced knockout (iKO) mice, one‐month‐old *Ogt* ^fl/fl^ Cre+ mice were administered tamoxifen (Sigma–Aldrich, St. Louis, MO) in corn oil by daily intraperitoneal injection at 50 µg g^−1^ body weight three times. After 50 days of normal feeding, mice were sacrificed and their lungs removed for downstream experiments. All animal studies were approved by the Animal Care and Use Committee of Nankai University, and mice were housed and operated in accordance with relevant regulations (2021‐SYDWLL‐000007).

### Cell Culture

MCF10A cells were purchased from the China Center for Type Culture Collection (CCTCC, Shanghai, China) and cultured in mammary epithelial cell growth medium (MEGM kit; Lonza/Clonetics, Basel, Switzerland, CC‐3150) with 100 ng mL^−1^ cholera toxin (Sigma–Aldrich, C8052). HEK293T cells were obtained from the American Type Culture Collection (ATCC, Manassas, VA) and were cultured in Dulbecco's modified Eagle medium (DMEM) with 10% fetal bovine serum (FBS; VivaCell Biotechnology, Baden‐Württemberg, Germany, C04001‐500). All cell types were cultured in a humidified incubator containing 5% CO_2_ at 37 °C.

### Embryo Collection and Early Embryonic Development

Eight‐week‐old C57BL/6J female mice were super‐ovulated by intraperitoneal injection of 10 IU pregnant mare serum gonadotropin (PMSG, SanSheng, Ningbo, China) followed by 10 IU human chorionic gonadotropin (hCG, ShuSheng)  48 h later. Super‐ovulated female mice were caged individually with one fertile male overnight and mating was confirmed by the presence of a vaginal plug. Embryos were obtained by flushing the fallopian tubes and uterine horns with M2 medium (M7167, Sigma–Aldrich). Embryos were cultured in KSOM medium (EmbryoMax KSOM, Sigma–Aldrich, MR‐121), and then 8‐cell stage embryos were treated with DMSO or OSMI‐1, respectively. The developmental potential and polarity of embryos were assessed by immunofluorescence microscopy.

### Micro‐CT

Mice were anesthetized by intraperitoneal injection with tribromoethanol (T48402, Sigma–Aldrich). Micro‐CT images were acquired using the SkyScan 1276 high‐resolution µCT system (Bruker, Billerica, MA) at 50 kVp, 180 µA, and 150 mGy. After radiographic data were acquired, images were reconstructed by 2D image analysis software provided by CTvox (SkyScan), and 3D images were reconstructed using CTVol (SkyScan).

### Antibodies and Beads

The following antibodies were used: anti‐OGT (ab96718, Abcam, Cambridge, UK); anti‐*O*‐GlcNAcylation (CTD110.6, 0 7764, Sigma–Aldrich); anti‐αSMA (A2547, Sigma–Aldrich); anti‐vimentin (V2258, Sigma—Aldrich); anti‐N‐cadherin (610 920, BD, East Rutherford, NJ); anti‐E‐cadherin (610 182, BD); anti‐PARD6B (sc166405, Santa Cruz Biotechnology, Dallas, TX); anti‐α‐tubulin (ab18251, Abcam); anti‐γ‐tubulin (ab11316, Abcam); anti‐β‐actin (ab8226, Abcam); anti‐GFP (11 814 460 001, Roche, Basel, Switzerland); anti‐HA (H3663, Sigma–Aldrich); anti‐Myc (m4439, Sigma–Aldrich); anti‐GST (G7781, Sigma–Aldrich); anti‐Ezrin (610 603, BD); anti‐PCNT (P3340‐50, Bethyl Laboratories); anti‐CDK5RAP2 (A300‐554A, Bethyl Laboratories); anti‐CEP152 (A302‐480A, Bethyl Laboratories, Montgomery, TX); anti‐CEP192 (A302‐324A, Bethyl Laboratories); anti‐PCM1 (sc398365, Santa Cruz); anti‐CEP131 (ab99379, Abcam). Alexa Fluor 488, 568, and 647 secondary antibodies were from Life Technologies (Waltham, MA). DAPI (D5942) and TRITC‐phalloidin (P1951) were purchased from Merck KGaA (Darmstadt, Germany). The following beads were used in immunoprecipitation analyses: anti‐GFP beads (ab193983, Abcam), anti‐HA agarose (A2095, Sigma–Aldrich), and Protein A/G agarose (20 422) and Ni‐NTA His‐binding agarose (70 666) from Thermo Fisher Scientific.

### Plasmids, RNAi, and Drugs

Mammalian expression plasmids for GFP‐OGT, HA‐OGT, HA‐PCM1, GPF‐PCM1, GPF‐CEP131, Flag‐CEP131, and Flag‐CEP131 truncations were generated by PCR. All constructs were confirmed by DNA sequencing. Plasmids were transfected into HEK293T cells using polyethylenimine (PEI, 23966‐1, Polysciences, Warrington, PA) and into MCF10A cells using Lipofectamine 3000 (L3000‐015, Invitrogen, Waltham, MA). Prokaryotic expression plasmids His‐GFP‐PCM1‐C, His‐GFP‐PCM1‐M, and His‐GFP‐PCM1‐N were described previously.^[^ ^23^ ^]^ The siRNAs were transfected using Lipofectamine RNAiMAX (Invitrogen). The sequences for OGT siRNAs were 5′‐GGAUGGAAUUCAUAUCCUU‐3′ (#1) and 5′‐AUACGAUGGCAUCUUCUGGUAACCC‐3′ (#2). OSMI‐1 (SML1621) and thiamet G (TMG, SML0244) were purchased from Sigma–Aldrich.

### Protein Purification and Liquid Droplet Formation

His‐GFP‐tagged Pcm1 N, Pcm1 M, and Pcm1 C were expressed in *E. coli* BL21(DE3) strain for 20 h at 16 °C in the presence of 1 mm IPTG. Bacteria were lysed in ice‐cold lysis buffer (50 mm NaH_2_PO_4_, 500 mm NaCl, 10 mm imidazole, 0.5% Triton X‐100, 10% glycerol, at pH 8.0) containing 1 mm PMSF, 1 mm DTT, and protease inhibitor cocktail using a high‐pressure homogenizer. Proteins were absorbed on Ni‐NTA beads, eluted using 50–300 mm imidazole, and dialyzed into the P buffer (20 mm Tris‐HCl, pH 8.0, 150 mm NaCl, 10% glycerol, and 1 mm DTT) at 4 °C overnight. They were further concentrated to 500 µm for His‐GFP‐Pcm1‐N and His‐GFP‐Pcm1‐C and 300 µm for His‐GFP‐Pcm1‐M using Amicon Ultra 30 K centrifugal filter devices (MilliporeSigma, Burlington, MA). Protein aliquots (5 or 10 µL) were snap‐frozen in liquid nitrogen and stored at −80 °C.

Five microliters of the proteins at varying concentrations, with or without the in vitro *O*‐GlcNAcylation system, were incubated at 37 °C for 2 h. After incubation, 5% polyethylene glycol 3350 (PEG3350, 202 398, Sigma–Aldrich) was added before incubating at 25 °C for 2 min. After incubation, 3 µL of each mixture were loaded onto a glass coverslip on a glass slide and imaged under a widefield fluorescent microscope (Zeiss LSM900) with 20× or 40× objectives or a 60× oil immersion objective. FRAP assays were performed following the FRAP wizard installed on a Zeiss LSM900 confocal microscope system.

### Immunofluorescence Microscopy

Cells grown on glass coverslips or frozen sections were fixed with 4% paraformaldehyde (PFA, P6148, Sigma–Aldrich) for 30 min and permeabilized in 0.5% Triton X‐100 (93 443, Sigma–Aldrich) for 15 min. Then, cells or frozen sections were blocked in 4% bovine serum albumin (BSA, A1128, Gentihold) in PBS for 1 h and then incubated with primary antibodies, secondary antibodies, and DAPI. Cells or frozen sections were examined with a Zeiss LSM710 confocal microscope. The fluorescent intensity was quantified by ImageJ. 3D images were reconstructed using Imaris Microscopy Image Analysis Software (Oxford Instruments, Abingdon, UK).

### Quantification of Centrosome Position and Cell Polarity

For cavity formation, images of MCF10A cells were acquired on a Zeiss LSM710 confocal microscope at 40× objective with a z step of 500 nm. For the determination of centrosome position, the angles formed between the cavity edge and the nucleus‐centrosome axis were quantified. The angles were measured with ImageJ, and the values were between 0° and 180°. The centrosome position distribution was then analyzed.

For micropatterns, single MCF10A cells were plated on H‐shaped micropatterns and cultured two daughter cells to grow to a full pattern. Image analysis for centrosome positioning was performed using a series of macros in ImageJ. In brief, nuclei in the DAPI channel were detected using image thresholding and object size criteria, and centrosomes were detected with similar thresholding and by using nuclei region of interest (ROI) as a spatial reference. In order to analyze cell polarity, a system of axes was established as shown in Figure 3M, including the nucleus‐nucleus axis (NN axis, also *x*‐axis) passing through the center of nuclei of cell doublets and an axis perpendicular to the NN axis (*y*‐axis). The polarity index was calculated as the length of the centrosome to the *y*‐axis (NC_x_) / nuclear radius (N_R_).^[^ ^24^ ^]^

### TEM Analysis

Fresh mouse lung tissues were fixed in 2.5% glutaraldehyde (G5882, Sigma–Aldrich), post‐fixed in 2% osmium tetroxide, dehydrated in graded ethanol and propylene oxide, embedded in Epon, and cured for 24 h at 60 °C. Ultrathin sections (50 nm) were placed onto 200 mesh copper grids and double stained with uranyl acetate and lead citrate before transmission electron microscopy analysis (Hitachi HT7700 Exalens, Hitachi Hi‐Tech, Tokyo, Japan).

### Immunoblotting and Co‐Immunoprecipitation

For immunoblotting, cells and tissues were lysed using RIPA cell lysis buffer (50 mm Tris–HCl, 1% Triton, 0.1% SDS, 1% sodium deoxycholate, 150 mm NaCl, and 1 mm EDTA at pH 7.5), and equal amounts of proteins were separated by SDS‐PAGE. For immunoprecipitation, cells were lysed using the immunoprecipitation lysis buffer (20 mm Tris–HCl, 150 mm NaCl, 1 mm EDTA, 1 mm EGTA, 1% NP‐40, 2.5 mm sodium pyrophosphate, and 10% glycerol at pH 7.5) and supplemented with protease inhibitor cocktail (Roche). Cell lysates were incubated with beads (primary antibodies were added when using Protein A/G beads) at 4 °C for 4 h, washed five times with the immunoprecipitation lysis buffer, and then subjected to immunoblotting. For sWGA pull‐down detection, cells were lysed using the immunoprecipitation lysis buffer and supplemented with the protease inhibitor cocktail. Cell lysates were incubated with sWGA beads at 4 °C for 4 h, washed five times with the immunoprecipitation lysis buffer, and then subjected to immunoblotting.

### *O*‐GlcNAc Enzymatic Labeling

Cells were lysed using the immunoprecipitation lysis buffer and immunoprecipitated using anti‐PCM1 or anti‐CEP131 antibody. The immunopurified PCM1 or CEP131 was enzymatically labeled with an azido‐containing nucleotide sugar analog (UDP‐GalNAz) using an engineered (1,4)‐galactosyltransferase (GalT1 Y289L) according to the Click‐iT *O*‐GlcNAc enzymatic labeling kit protocol (Invitrogen) and conjugated with an alkyne‐biotin compound as per the Click‐iT protein analysis detection kit protocol (Invitrogen). Biotin‐labeled samples were subsequently probed with STV‐HRP. Control experiments were performed in parallel in the absence of GalT1 Y289L.

### In Vitro *O*‐GlcNAcylation

In vitro *O*‐GlcNAcylation was performed with 1 µm target protein as standard.^[^ ^25^ ^]^ Reaction mixtures containing 1 µm purified indicated protein, 0.125 µm purified OGT protein, and 10 µm UDP‐GlcNAc (Sigma) in a buffer of 50 mm Tris/HCl (pH 7.5) and 1 mm DTT were incubated at 37 °C for 1 h. Then, the protein *O*‐GlcNAcylation test was performed.

### Mass Spectrometry

To identify OGT‐binding proteins, the anti‐OGT antibody was used to immunoprecipitate endogenous OGT from the MCF10A cell lysate. The immunoprecipitated proteins were subjected to SDS‐PAGE and Coomassie blue staining. Then the stained gel was subjected to mass spectrometry by PTM Biolabs. In brief, the eluted proteins were digested to peptides and subjected to tandem mass spectrometry using Q‐Exactive Plus and Orbitrap Elite systems (Thermo Fisher Scientific) at a resolution of 70 000. Proteome Discoverer 1.3 (Thermo Fisher Scientific) was used as the peaklist‐generating software and the search engine.

### Live‐Cell Imaging

MCF10A cells were seeded in a live cell culture dish and grown to 40–60% confluency, and then cells were transfected with GFP‐PCM1 or GFP‐CEP131 plasmids. Cells were then treated with DMSO or OSMI‐1 and the time‐lapse images were captured by confocal fluorescence microscopy (Zeiss LSM900).

### Statistical Analysis

Data were analyzed using the two‐tailed unpaired Student's *t*‐test to compare two groups or ANOVA to compare more than two groups and are expressed as mean ± SD. *p*‐values less than 0.05 were considered significant. Quantitative statistics and mapping were completed using ImageJ software and GraphPad Prism software (GraphPad Software, La Jolla, CA).

## Conflict of Interest

The authors declare no conflict of interest.

## Author Contributions

F.Y. and S.Y. contributed equally to this work. F.Y. designed experiments, conducted experiments, analyzed data, and wrote the manuscript. S.Y. conducted experiments, analyzed data, and wrote the manuscript. H.N., D.H., X.W., M.Y., X.Z., Y.C., Y.P., and D.A. conducted experiments. D.Li, D.Liu, L.L., L.P., Q.C., and X.Z. provided guidance for the experiments. J.Z. supervised the project and revised the manuscript.

## Supporting information

Supporting Information

## Data Availability

The data that support the findings of this study are available from the corresponding author upon reasonable request.
