# Supplementary material for: O‐GlcNAcylation Regulates Centrosome Behavior and Cell Polarity to Reduce Pulmonary Fibrosis and Maintain the Epithelial Phenotype
Source: Adv Sci (Weinh). 2023 Nov 14;10(36):2303545. doi: 10.1002/advs.202303545 (PMC10754140; doi:10.1002/advs.202303545)
Supplement: Supplementary file 1 — Supporting Information [file ADVS-10-2303545-s001.pdf]

ADVANCED SCIENCE
Open Access

Supporting Information

for *Adv. Sci.*, DOI 10.1002/advs.202303545

O-GlcNAcylation Regulates Centrosome Behavior and Cell Polarity to Reduce Pulmonary Fibrosis and Maintain the Epithelial Phenotype

Fan Yu, Song Yang, Hua Ni, Dai Heng, Xuemei Wu, Mulin Yang, Xinming Zhang, Yuxin Cao, Yandong Pei, Di An, Dengwen Li, Dayong Liu, Lin Liu, Leiting Pan, Quan Chen, Xueliang Zhu and Jun Zhou**

SUPPLEMENTARY FIGURES AND FIGURE LEGENDS

Fig. S1

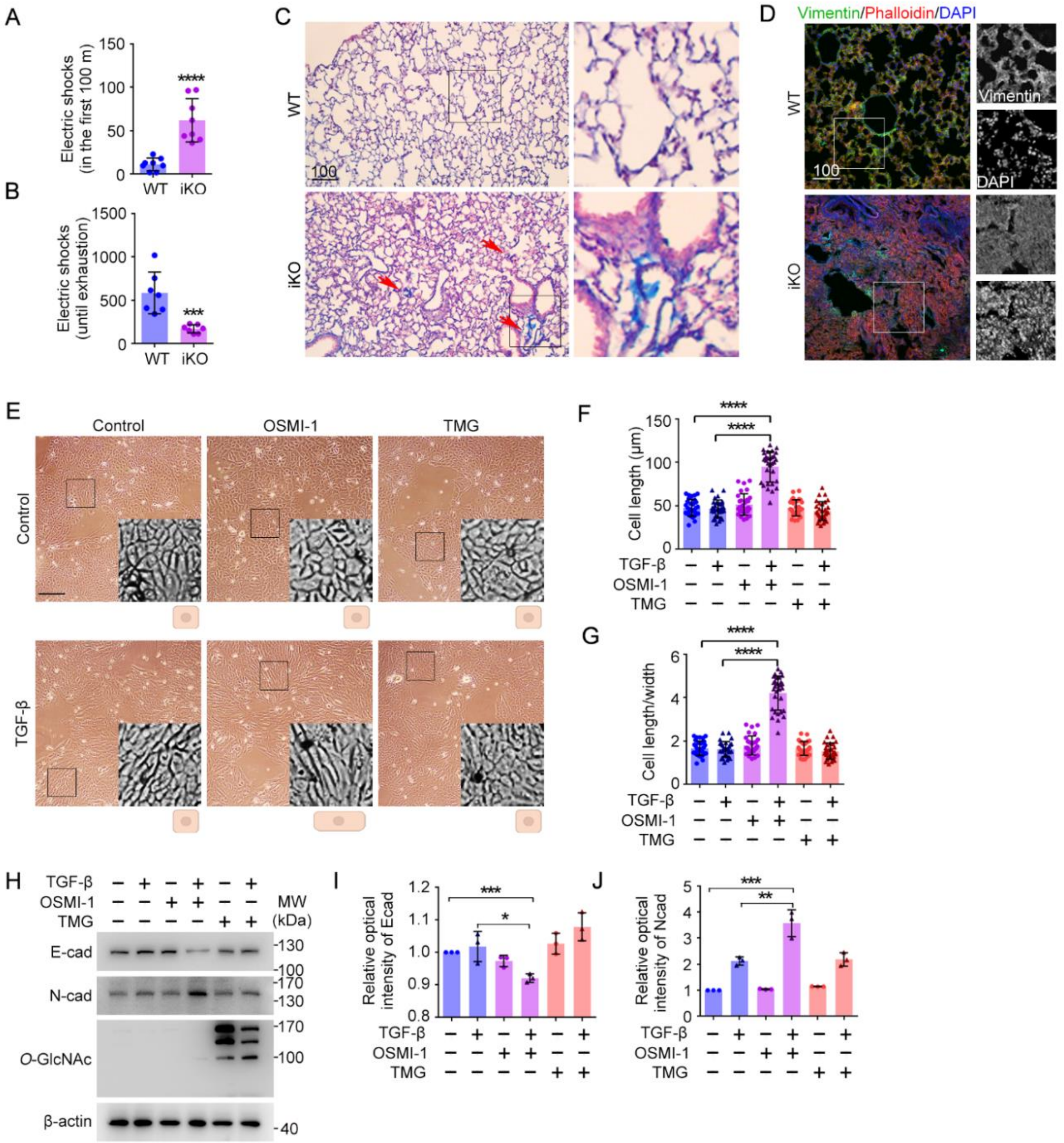

Fig. S1. (**A**, **B**) The physical ability of *Ogt* iKO mice was assessed on a treadmill, including the times of electric shocks needed to stimulate running over the first 100 meters (A, n = 8 mice) and the total electric shocks from the beginning until exhaustion (B, n = 8 mice). (**C**) Masson trichrome staining of lung sections from WT and *Ogt* iKO mice. (**D**) Lung sections from WT and *Ogt* iKO mice were subjected to immunofluorescence microscopy with antibodies targeting vimentin. (**E-G**) MCF10A cells were treated with DMSO (Control), OSMI-1, or TMG with/without TGF-β for 24 h and subjected to microscopy imaging. The length (F, n = 30 cells) and the ratio of cell length:width (G, n = 30 cells) of each cell were quantified using ImageJ. (**H-J**) MCF10A cells were treated with DMSO (Control), OSMI-1, or TMG with/without TGF-β for 24 h and subjected to immunoblotting with the indicated antibodies. The relative intensity of E-cadherin (I) and N-cadherin (J) were quantified using ImageJ. All experiments were repeated at least three times. Scale bars are 10 unless specifically indicated (μm). **P* < 0.05, ***P* < 0.01, ****P* < 0.001, *****P* < 0.0001. Error bars indicate SD.

Fig. S2

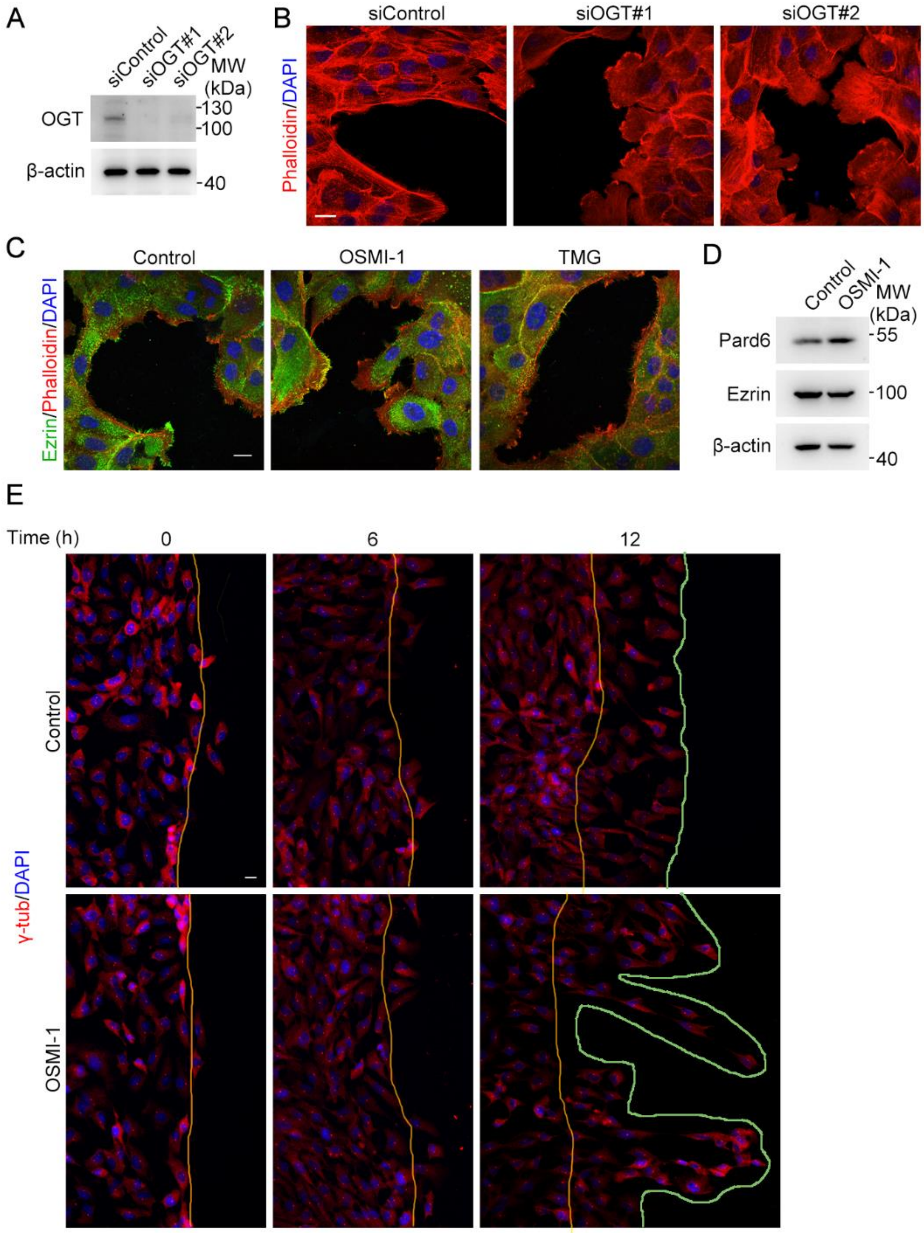

Fig. S2. (**A**) MCF10A cells were transfected with control or OGT siRNA for 48 h and then subjected to immunoblotting with the indicated antibodies. (**B**) MCF10A cells were transfected with control or OGT siRNA for 48 h and then subjected to immunofluorescence microscopy with phalloidin. **(C)** MCF10A cells were treated with DMSO (Control), OSMI-1, or TMG for 24 h and then subjected to immunofluorescence microscopy with antibodies targeting Ezrin and phalloidin. (**D**) MCF10A cells were treated with DMSO (Control) or OSMI-1 for 24 h and then subjected to immunoblotting with the indicated antibodies. (**E**) RPE1 cells were scratched along the orange line and treated with DMSO (Control) or OSMI-1 for the indicated time and then subjected to immunofluorescence microscopy with antibody targeting γ-tubulin. All experiments were repeated at least three times. Scale bars, 10 μm.

Fig. S3

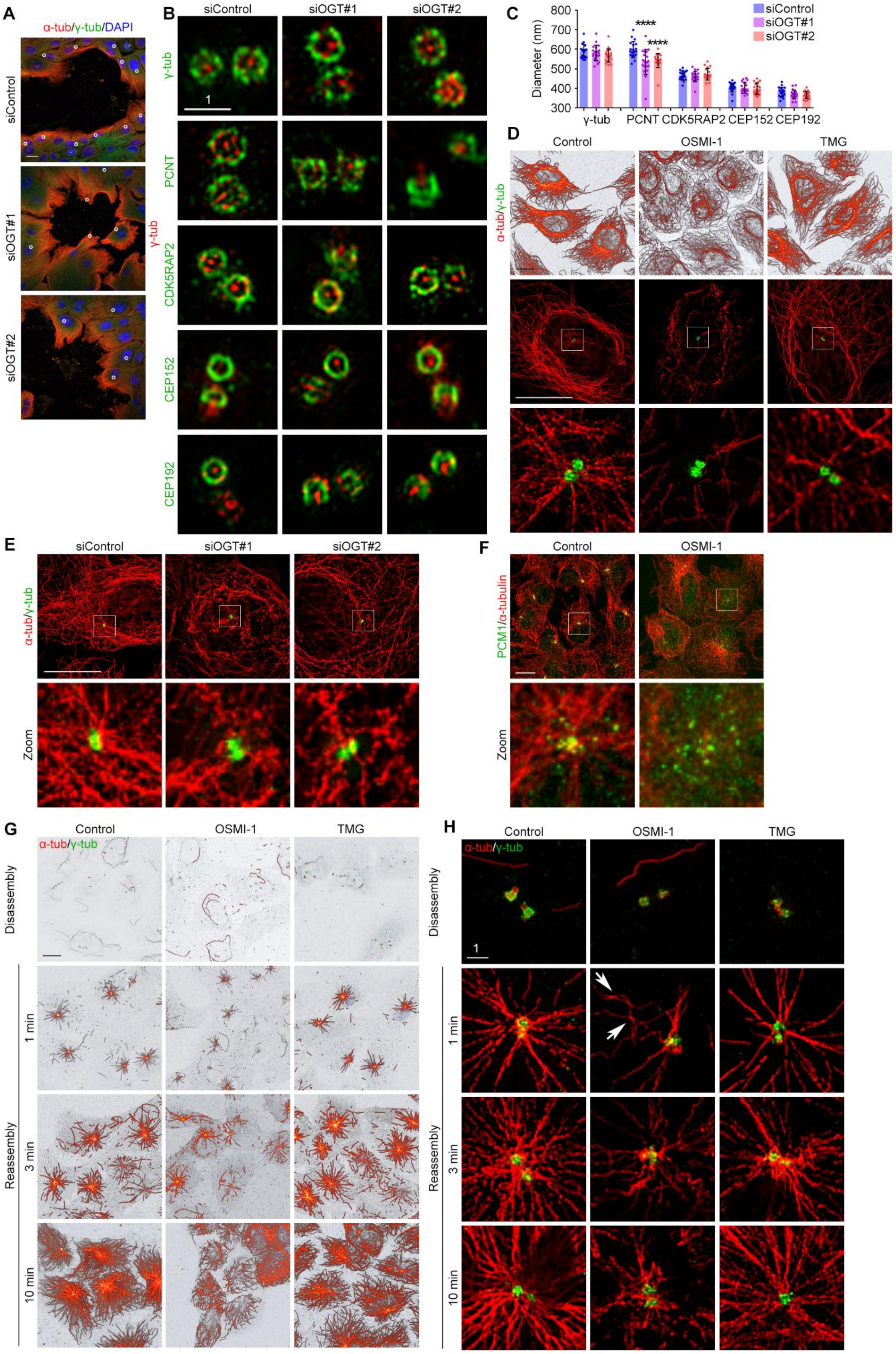

A
α-tub/γ-tub/DAPI
siControl
siOGT#1
siOGT#2
B
siControl
siOGT#1
siOGT#2
γ-tub
PCNT
CDK5RAP2
CEP152
CEP192
γ-tub
1
C
Diameter (nm)
800
700
600
500
400
300
****
****
siControl
siOGT#1
siOGT#2
γ-tub
PCNT
CDK5RAP2
CEP152
CEP192
D
Control
OSMI-1
TMG
α-tub/γ-tub
E
siControl
siOGT#1
siOGT#2
α-tub/γ-tub
Zoom
F
Control
OSMI-1
PCM1/α-tubulin
Zoom
G
Control
OSMI-1
TMG
α-tub/γ-tub
Disassembly
Reassembly
1 min
3 min
10 min
H
Control
OSMI-1
TMG
α-tub/γ-tub
Disassembly
1
Reassembly
1 min
3 min
10 min

Fig. S3. (**A**) MCF10A cells were transfected with control or OGT siRNA for 48 h and then subjected to immunofluorescence microscopy with antibodies targeting α-tubulin and γ-tubulin. Centrosomes are highlighted with white circles. (**B, C)** MCF10A cells were transfected with control or OGT siRNA for 48 h and then subjected to immunofluorescence microscopy with the indicated antibodies (B). The diameter of each ring was quantified using ImageJ (C, n = 20 cells). **(D)** MCF10A cells were treated with DMSO (Control), OSMI-1, or TMG for 24 h and then subjected to immunofluorescence microscopy with antibodies targeting α-tubulin and γ-tubulin. (**E**) U2OS cells were transfected with control or OGT siRNA for 48 h and then subjected to immunofluorescence microscopy with antibodies targeting α-tubulin and γ-tubulin. (**F**) U2OS cells were treated with DMSO (Control) or OSMI-1 for 24 h and then subjected to immunofluorescence microscopy with antibodies targeting α-tubulin and PCM1. **(G, H)** MCF10A cells were treated with DMSO (Control), OSMI-1, or TMG for 24 h and the microtubules were fully disassembled on ice for 30 min. Then, the microtubules were reassembled for the indicated times and subjected to immunofluorescence microscopy with antibodies targeting α-tubulin and γ-tubulin. All experiments were repeated at least three times. Scale bars are 10 unless specifically indicated (μm). **** $P < 0.0001$. Error bars indicate SD.

Fig. S4

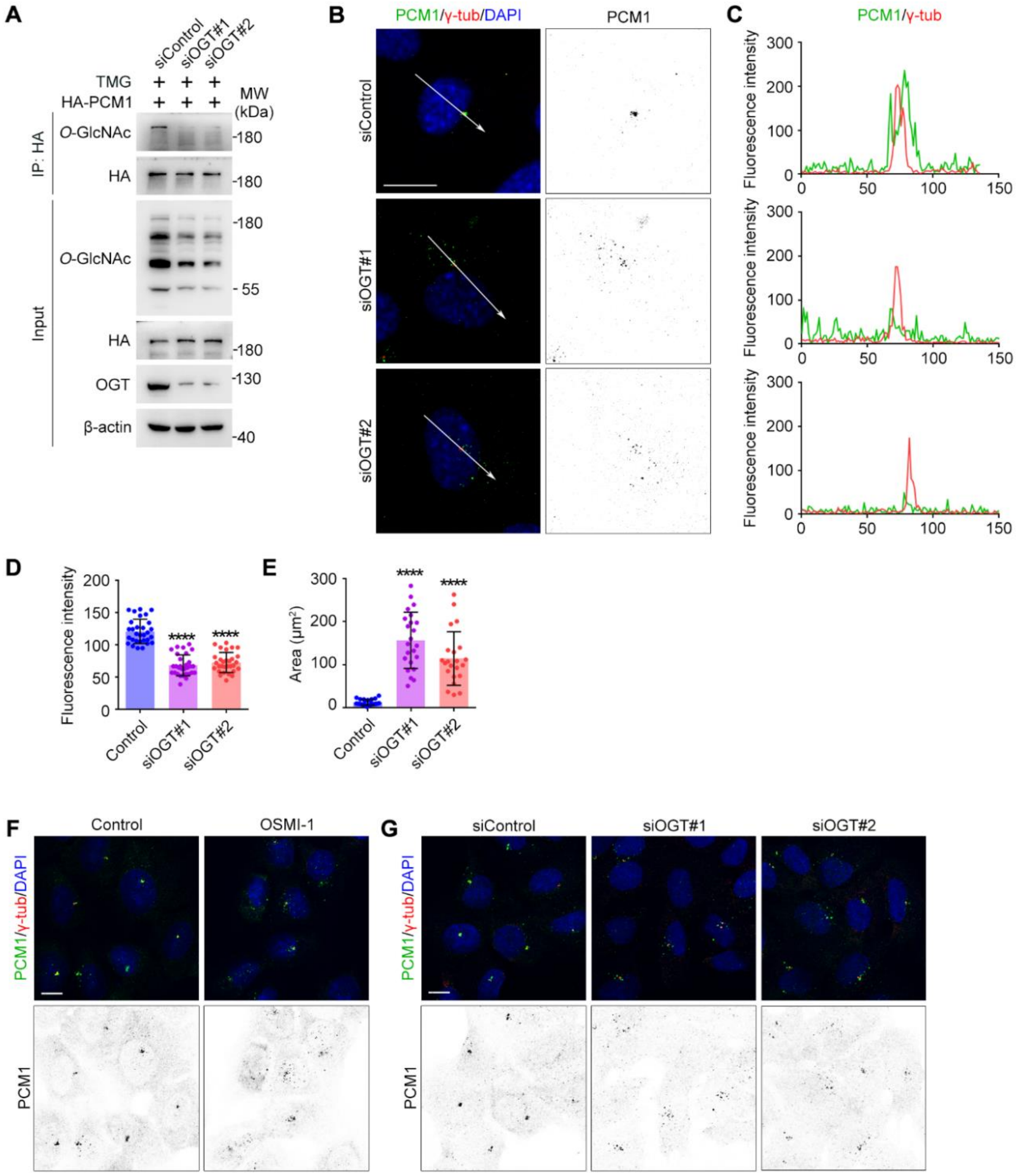

Fig. S4. (**A**) 293T cells were transfected with control or OGT siRNA for 24 h and then transfected with HA-PCM1 plasmids and treated with TMG for another 24 h. Cell lysates were subjected to immunoprecipitation. (**B-E**) MCF10A cells were transfected with control or OGT siRNA for 48 h and then subjected to immunofluorescence microscopy with antibodies targeting PCM1 and γ-tubulin (B). The fluorescence intensity of PCM1 along the arrow was assessed by ImageJ (C). The fluorescence intensity of PCM1 around the centrosome (D, n = 30 cells) and the distribution area of PCM1 (E, n = 30 cells) was quantified by ImageJ. (**F**) U2OS cells were treated with control or OSMI-1 for 24 h and then subjected to immunofluorescence microscopy with antibodies targeting PCM1 and γ-tubulin. (**G**) U2OS cells were transfected with control or OGT siRNA for 48 h and then subjected to immunofluorescence microscopy with antibodies targeting PCM1 and γ-tubulin. All experiments were repeated at least three times. Scale bar, 10 μm. **** $P < 0.0001$. Error bars indicate SD.

Fig. S5

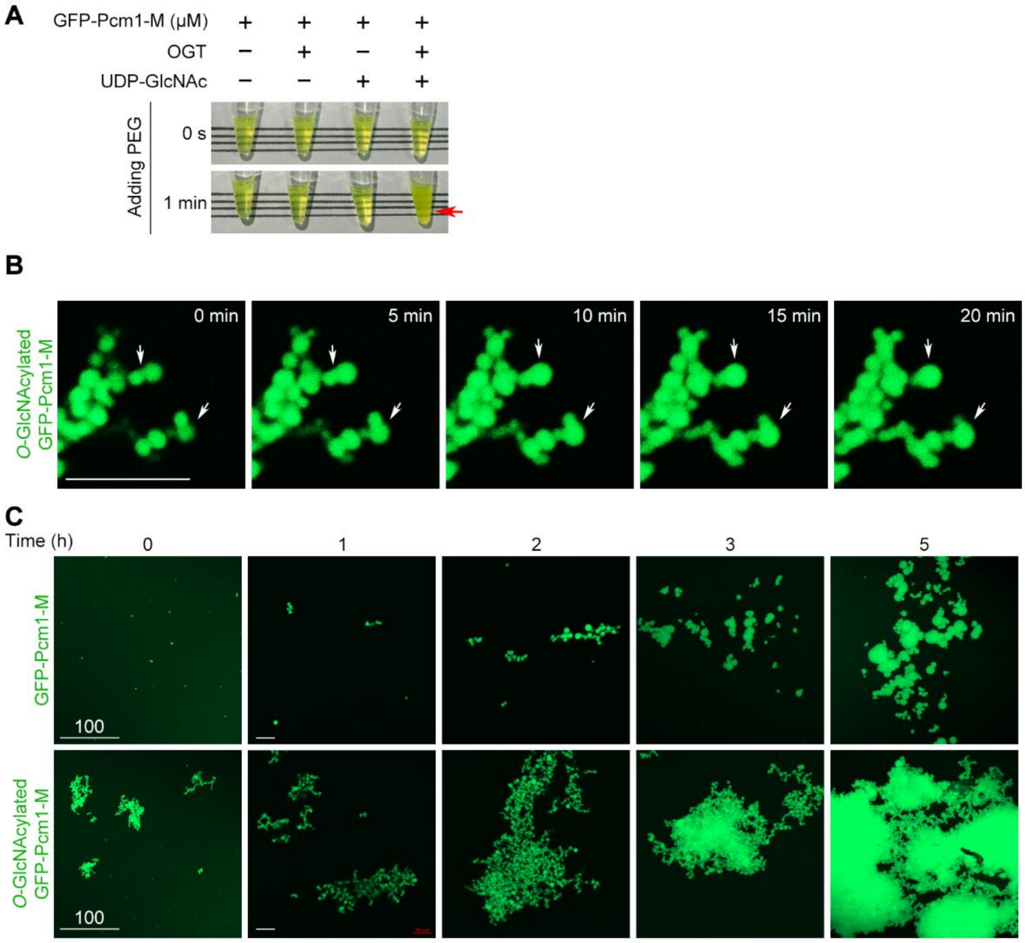

Fig. S5. (**A**) Purified His-GFP-Pcm1M was subjected to *in vitro O*-GlcNAcylation assay with or without UDP-GlcNAc/OGT as indicated. The reaction mixtures were then treated with PEG and imaged. (**B**) Purified His-GFP-Pcm1-M was subjected to *in vitro O*-GlcNAcylation assay and the mixtures were then treated with PEG and live imaged with fluorescence microscopy. (**C**) Purified His-GFP-Pcm1-M was subjected to *in vitro O*-GlcNAcylation assay with or without UDP-GlcNAc and OGT. The mixtures after reaction were placed at room temperature for the indicated time and then subjected to fluorescence microscopy. All experiments were repeated at least three times. Scale bars are 10 unless specifically indicated (μm).

Fig. S6

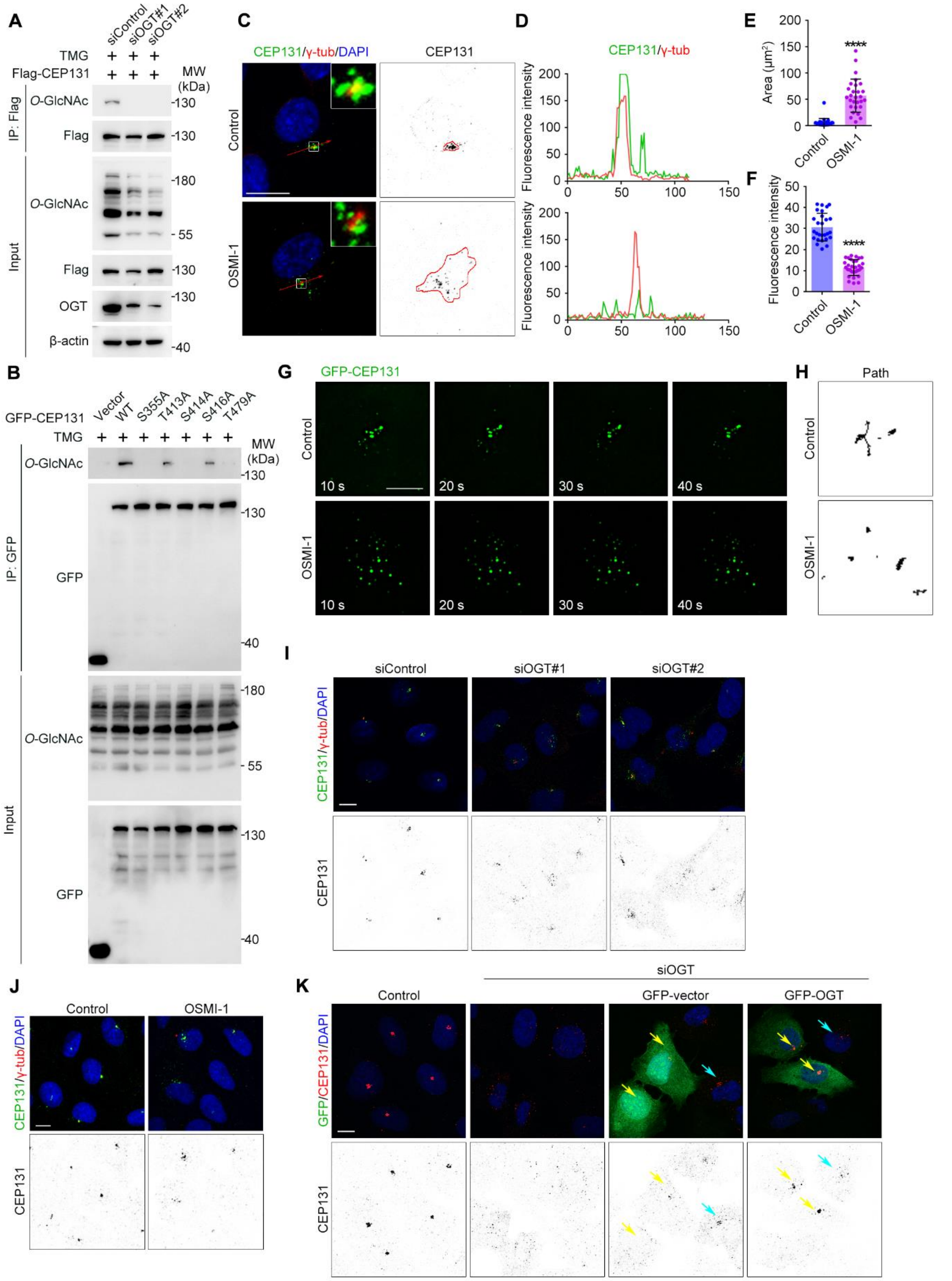

Fig. S6. (**A**) 293T cells were transfected with control or OGT siRNA for 24 h and then transfected with Flag-CEP131 plasmids and treated with TMG for another 24 h. Cell lysates were subjected to immunoprecipitation. (**B**) 293T cells were transfected with GFP-vector, GFP-CEP131, or the single-site mutants of GFP-CEP131, treated with TMG for 24 h, and subjected to immunoprecipitation. (**C-F**) MCF10A

cells were treated with DMSO (Control) or OSMI-1 for 24 h and then subjected to immunofluorescence microscopy with antibodies targeting CEP131 and γ-tubulin (C). The fluorescence intensity of CEP131 along the red arrow in panel C was assessed using ImageJ (D). The distribution area of CEP131 (E, n = 30 cells) and the fluorescence intensity of CEP131 around the centrosome (F, n = 30 cells) was quantified by ImageJ. (**G, H**) MCF10A cells were transfected with GFP-CEP131 plasmid, treated with DMSO (Control) or OSMI-1 for 24 h, and then subjected to live cell imaging (G). The moving paths of GFP-CEP131 were assessed using ImageJ (H). (**I**) U2OS cells were transfected with control or OGT siRNA for 48 h and then subjected to immunofluorescence microscopy with antibodies targeting CEP131 and γ-tubulin. (**J**) U2OS cells were treated with DMSO (Control) or OSMI-1 for 24 h and then subjected to immunofluorescence microscopy with antibodies targeting CEP131 and γ-tubulin. (**K**) MCF10A cells were transfected with control or OGT siRNA for 24 h and then transfected with GFP-vector or GFP-OGT plasmids for another 24 h as indicated. The cells were subjected to immunofluorescence microscopy with antibody targeting CEP131. Cells successfully transfected with plasmids were indicated by yellow arrows, while cells without plasmid transfection were indicated by blue arrows. All experiments were repeated at least three times. Scale bars, 10 μm. **** $P < 0.0001$. Error bars indicate SD.
